# Radical electron-induced cellulose-semiconductors

**DOI:** 10.1038/s41598-024-59499-1

**Published:** 2024-04-15

**Authors:** Mikio Fukuhara, Tomonori Yokotsuka, Tetsuo Samoto, Masahiko Kumadaki, Mitsuhiro Takeda, Toshiyuki Hashida

**Affiliations:** 1 New Industry Creation Hatchery Center, Tohoku University, Aoba, Sendai 980-8579 Japan; 2 Technical Section, Semilab Japan KK, Yokohama, 222-0033 Japan; 3 National Institute of Technology, Sendai College, Natori, 981-1239 Japan

**Keywords:** Biophysics, Biotechnology, Molecular biology, Energy science and technology, Materials science, Nanoscience and technology

## Abstract

Bio-semiconductors are expected to be similar to organic semiconductors; however, they have not been utilized in application yet. In this study, we show the origin of electron appearance, N- and S-type negative resistances, rectification, and switching effects of semiconductors with energy storage capacities of up to 418.5 mJ/m^2^ using granulated amorphous kenaf cellulose particles (AKCPs). The radical electrons in AKCP at 295 K appear in cellulose via the glycosidic bond C_1_–O_1_^·^–C_4_. Hall effect measurements indicate an *n*–type semiconductor with a carrier concentration of 9.89 × 10^15^/cm^3^, which corresponds to a mobility of 10.66 cm^2^/Vs and an electric resistivity of 9.80 × 10^2^ Ωcm at 298 K. The conduction mechanism in the kenaf tissue was modelled from AC impedance curves. The light and flexible cellulose-semiconductors may open up new avenues in soft electronics such as switching effect devices and bio-sensors, primarily because they are composed of renewable natural compounds.

## Introduction

Almost all organic semiconductors currently used as part of semiconductor materials are π-conjugated oligomers and polymers^1–4^. However, π-conjugated polymers are lacking in natural bio-compounds. Studies on naturally occurring bio-semiconductors are scarce^5^. We reported an *n*–type bio-semiconductor based on an amorphous kenaf cellulose nanofibre^6^ exhibiting rectification, *n*–type negative resistance, and DC/AC conversion. However, owing to their thixotropic properties that create aggregates containing entangled pores, producing dense films using long fibres with an aspect ratio of more than 100 is extremely challenging^7^. In this study, a prototype film composed of amorphous kenaf cellulose particles (AKCPs), which were defibrillated and milled to a diameter of ~ 11 nm, was produced, and the electronic properties of AKCPs were evaluated. Subsequently, electron spin resonance (ESR) measurements^8,9^, the only means of observing radicals in organic materials, were performed at 295 K to investigate the origin of electrons in the *n*–type semiconductors. Furthermore, electron mobility was quantified via Hall measurements. An analysis of the AC impedance elucidated the tissue-dependent conduction mechanism of AKCPs. Recently, compound semiconductor devices for the skin^10^, stretchable electronics^11^ and function of the retina^12^ with properties similar to those of bio-semiconductors, have been reported.

## Results and discussion

### Semiconducting characteristics with electric storage

**Figure 1:**
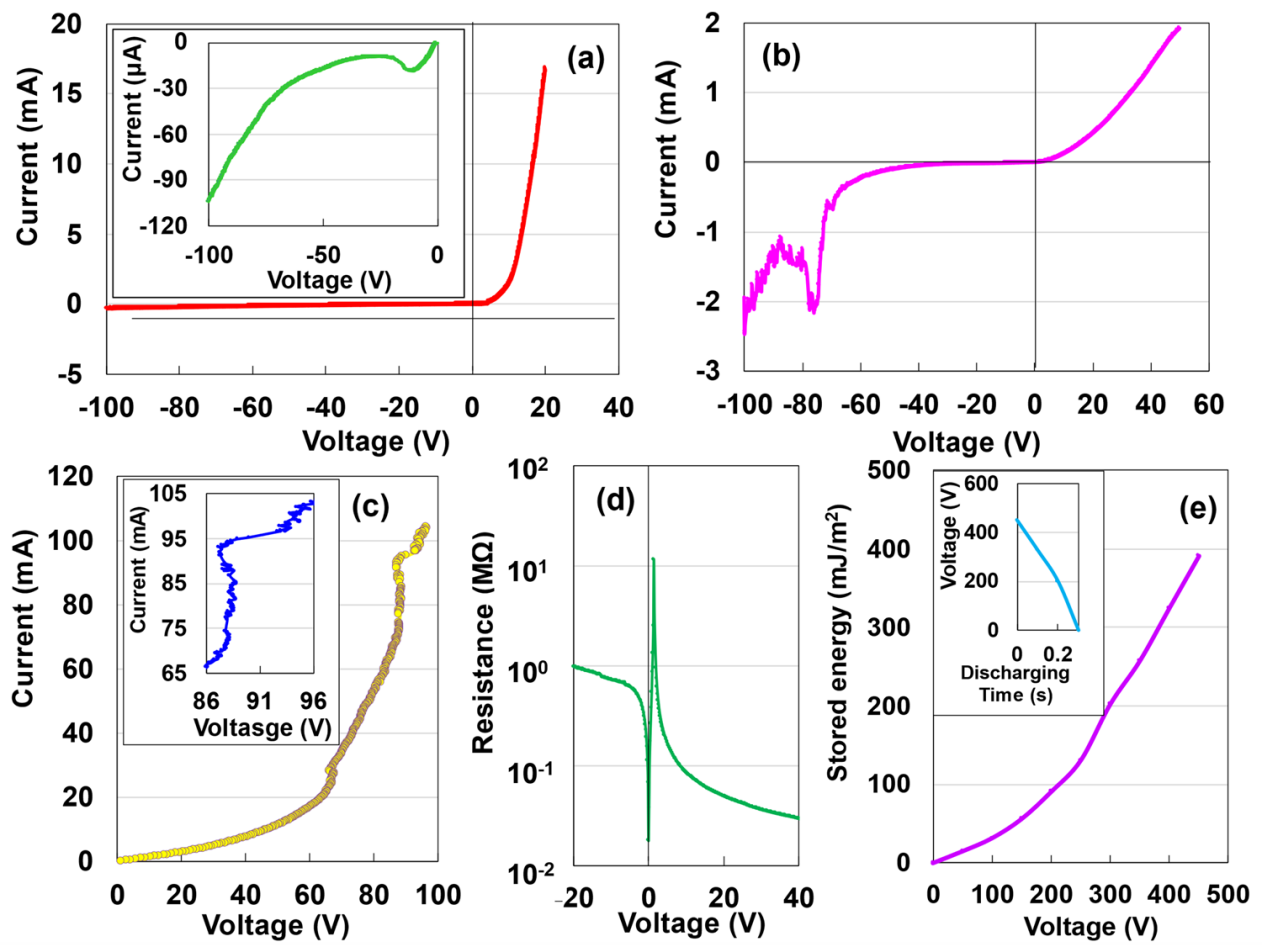
Voltage-controlled* I–V* characteristics of AKCPs with a thickness of 14 (**a**), 25 (**b**), and 12 µm (**d**) at a sweep rate of 51.5 V/s. (**c**) *I–V* characteristics in a current-controlled measurement at 0.105 A for AKCPs with a thickness of 19 μm. Inset of (c) shows an enlarged S-type figure. (**e**) Discharging behaviour of the AKCP device with a thickness of 101 µm for a constant current of 1 μA after 2 mA*–*10 V charging for 5 s. Inset of (**e**) shows discharging behaviour.

The DC measurement method was used to determine the voltage-controlled *I–V* characteristics of AKCPs for a sample with a thickness of 14 µm at 298 K within the current range of 0*–*20 mA and voltage range of –100 to 40 V (Fig. 1a). The *I–V* curve exhibits a clear forward rectification effect. Figure 1b illustrates the *I–V* characteristics for a sample thickness of 25 µm. A clear N-type negative resistance appears between approximately *–*87 V and *–*71 V, as well as a small N-type negative resistance (inset of Fig. 1a). In excess of 0 V, an increase is observed in the current value in the forward direction. Meanwhile, the* I–V* characteristics of a sample with a thickness of 19 µm in a current-controlled measurement at 0.105 A are shown in Fig. 1c, where the current value increases sharply from approximately 88 V during an increase from 0 to 100 V. The enlarged curve shown in the inset of Fig. 1c shows an* S*-type negative resistance, as in the Ni–Nb–Zr–H amorphous alloy^13^. Based on the semiconducting theory, the negative resistance characteristics are classified into static negative resistance characteristics, such as those of tunnel diodes and thyristors^14^, and dynamic negative resistance characteristics considering the carrier transfer time and material band structure specificity, such as those of impact avalanche transit diodes and Gunn diodes^15^. This study considers the latter (see Supplementary Information (SI). S9). The semiconductor properties of AKCPs obtained from the aforementioned experiments are shown in the SI, Fig. S4, which shows a Schottky junction* n*–type semiconductor^16^. The observed S-type negative resistance (Fig. 1c), in addition to the N-type negative properties (Fig. 1b), may be attributed to the kenaf becoming granular instead of fibrous. Figure 1d presents the *R–V* characterisation on a logarithmic scale from *–*20 to 40 V for AKCPs with a thickness of 12 μm. The *R–V* curve exhibits a three-field change in magnitude between 0 and 10 V, indicating a switching effect. In contrast, a specimen with a thickness of 101 µm exhibits a storage effect (Fig. 1e) that is not observed in the case of amorphous kenaf cellulose nanofibres^17^. The storage capacity increases with voltage, and a storage capacity of 418.5 mJ/m^2^ is obtained at 450 V. The discharge curve at 450 V is shown in the inset. This value is less than half the values for amorphous kenaf cellulose nanofibres^18^ and amorphous alumina (AAO)^19^ (1416.7 and 1710.3 mJ/m^2^, respectively). These phenomena may be attributed to an increase in the capacitance of the AKCPs with increasing thickness. The analysis results of trace impurities in the AKCPs are presented in Table S1. However, the effect of trace impurities on semiconductor properties is currently unclear. The effects of bound water will be addressed in a subsequent study.

### Origin of radical electrons derived from cellulose structure

**Figure 2:**
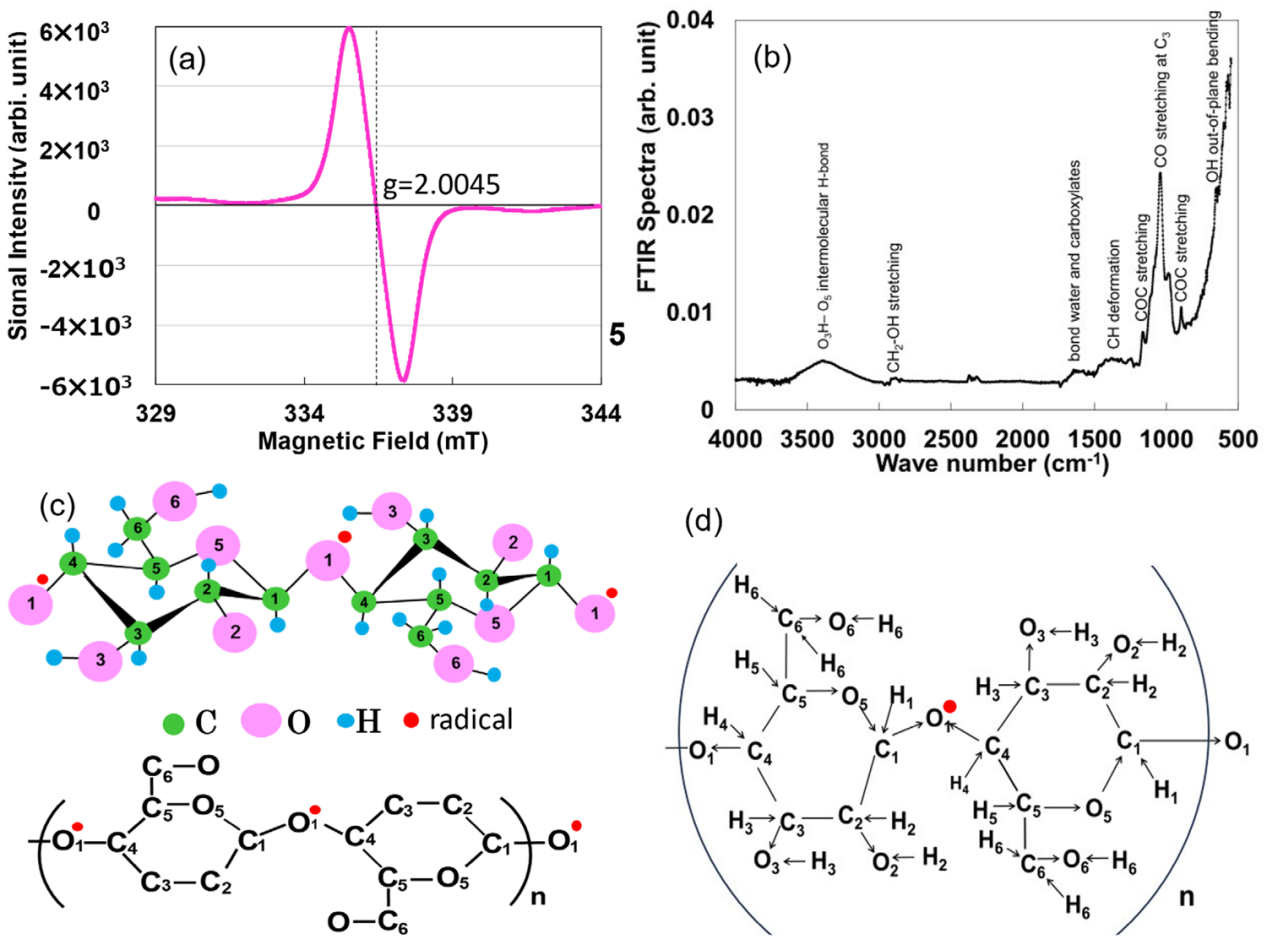
(**a**) Singlet symmetrical ESR spectra at 298 K. (**b**) ATR-FTIR spectroscopic analysis at 298 K for the AKCPs. (**c**) Cellulose comprising (C_6_H_10_O_5_)_n_ with the green, pink, and light-blue dots representing carbon, oxygen, and hydrogen atoms, respectively. (**d**) Electron-induced effects for cellulose structure.

The results of the ESR measurements are displayed in Fig. 2a. The curve obtained at 295 K is the isotropic peak of a singlet. As the unpaired electrons shown in the ESR spectrum are extremely sensitive to the molecular arrangement around the electrons, the g-value of the ESR signal presents a decisive guideline for identifying the organic radical species^20^. Based on the molecular structure of cellulose (C_6_H_10_O_5_)n, the g-values of 2.004 and 2.009 correspond to the alkoxy radical CO^·^^21^. In this study, we identify the position of the radical group for the cellulose molecule relative to the radical electron, which is the origin of electron conductivity. The alkoxy groups generated at the side chain Cs (C_2_, C_3_, C_6_) are more reactive than the alkoxy group generated at C_1_ on the main chain and are rapidly deactivated by secondary reactions^22^; therefore, the C_2_–O_2_, C_3_–O_3_, and C_6_–O_6_ groups in Fig. 2c are excluded as radical electron candidates. The radical electrons in the AKCPs appear in cellulose via the glycosidic bond between the two glucose units and O_1_. Attenuated total reflection-Fourier transform infrared (ATR-FTIR) measurements were performed to confirm the existence of glycosidic bonds. The results are shown in Fig. 2b. Peaks of COC stretching motion are observed at 1184 and 884 cm^−1^ (Ref.^23^). Considering the electronegativity values of 2.20, 2.55, and 3.44 for H, C, and O, respectively, the electron-induced effects are depicted in Fig. 2d when applied to cellulose molecules (see S7 in SI for details). The locations of the appearance of the radical electrons obtained from ESR are shown in Fig. 2c, as C_1_–O_1_^·^–C_4_. The electrons induced in conventional organic semiconductors are π-electrons from the C=C double bond^24^. The Hall coefficient measurements showed an electron mobility of 10.66 cm^2^/Vs, a carrier density of –9.89 × 10^15^ (1/cm^3^), and an electric resistivity of 9.80 × 10^2^ Ωcm at 298 K. The mobility is two orders higher than 0.5–1.0 cm^2^/Vs^25^ for amorphous Si and 0.08–2.5 cm^2^/Vs^26,27^ for π-conjugated organic semiconductors. However, Farka et al.^28^ reported a relative high value of 40.8 cm^2^/Vs in p-doped polyethylene-(3,4-dioxythiophene) (PEDOT: sulfate).

### Complex evaluation of *I–V* characteristics

**Figure 3:**
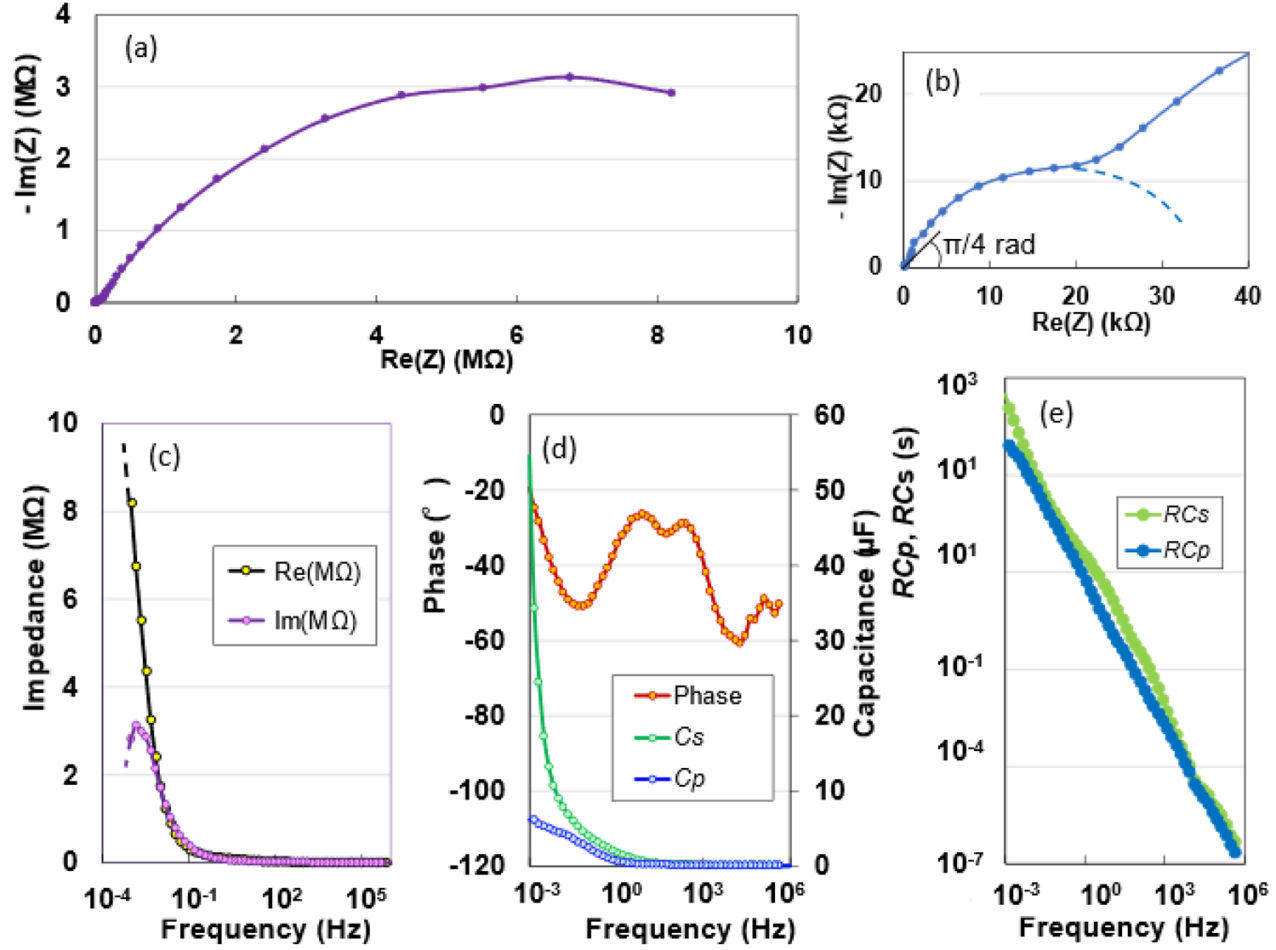
(**a**,**b**) Nyquist plots as a function of frequency for the AKCP device. (**c**) Frequency dependence of real and imaginary impedances, (**d**) phase angle, and series and parallel capacitances. (**e**) Frequency dependence of *RC*s and *RCp*.

To non-destructively analyse the electronic contribution of the sample, its AC impedance was measured from 1 mHz to 1 MHz. The Nyquist diagram of the impedance data, corresponding to 14 μm in Fig. 1a, is illustrated in Fig. 3a and b. The impedance of the AKCPs with respect to frequency exhibits a linear slope with a change of π/4 rad, as shown in Fig. 3b, and a combined pattern of two semicircles. The π/4 rad region (Warburg region) can be attributed to the porous sample^6,29,30^. The two semicircles represent a tissue composed of two fibres, such as the bast and core in kenaf^31^. The peak frequency *f*_*max*_ of the semicircle is 1.48 mHz; therefore, a relaxation time of 107.6 s can be calculated using the relationship *RC*_*total*_ = *1/(2πf*_*max*_*)*. In the low-frequency region of the Bode diagram shown in Fig. 3c, the real impedance increases sharply to 8 MΩ, whereas the imaginary impedance peaks at 1.48 mHz and decreases at relatively low frequencies. The 1.48-mHz peak corresponds to dielectric dispersion owing to interfacial polarisation in the low-frequency range^32^. In the phase angle diagram of Fig. 3d, the capacitance behaviour for frequencies lower than 0.073 Hz (near zero phase angle) is clearly similar to that of a parallel *RC* circuit. The series capacitance *C*_*s*_ and parallel capacitance *C*_*p*_ increase as the frequency decreases; however, the increase in *C*_*s*_ is relatively rapid. *C*_*s*_ plays a vital role in determining the DC *I-V* characteristics of the sample. In the relationship between the time constant *RC* and frequency, as shown in Fig. 3e, in both logarithmic displays, the time constants *RC*_*s*_ and *RC*_*p*_ increase almost linearly with decreasing frequency; *RC*_*s*_ and *RC*_*p*_ at 1 mHz are 448 and 50 s, respectively. A larger duration (from 0.1 s to a few hours) is required for practical use.

### Mechanism of electron conduction

**Figure 4:**
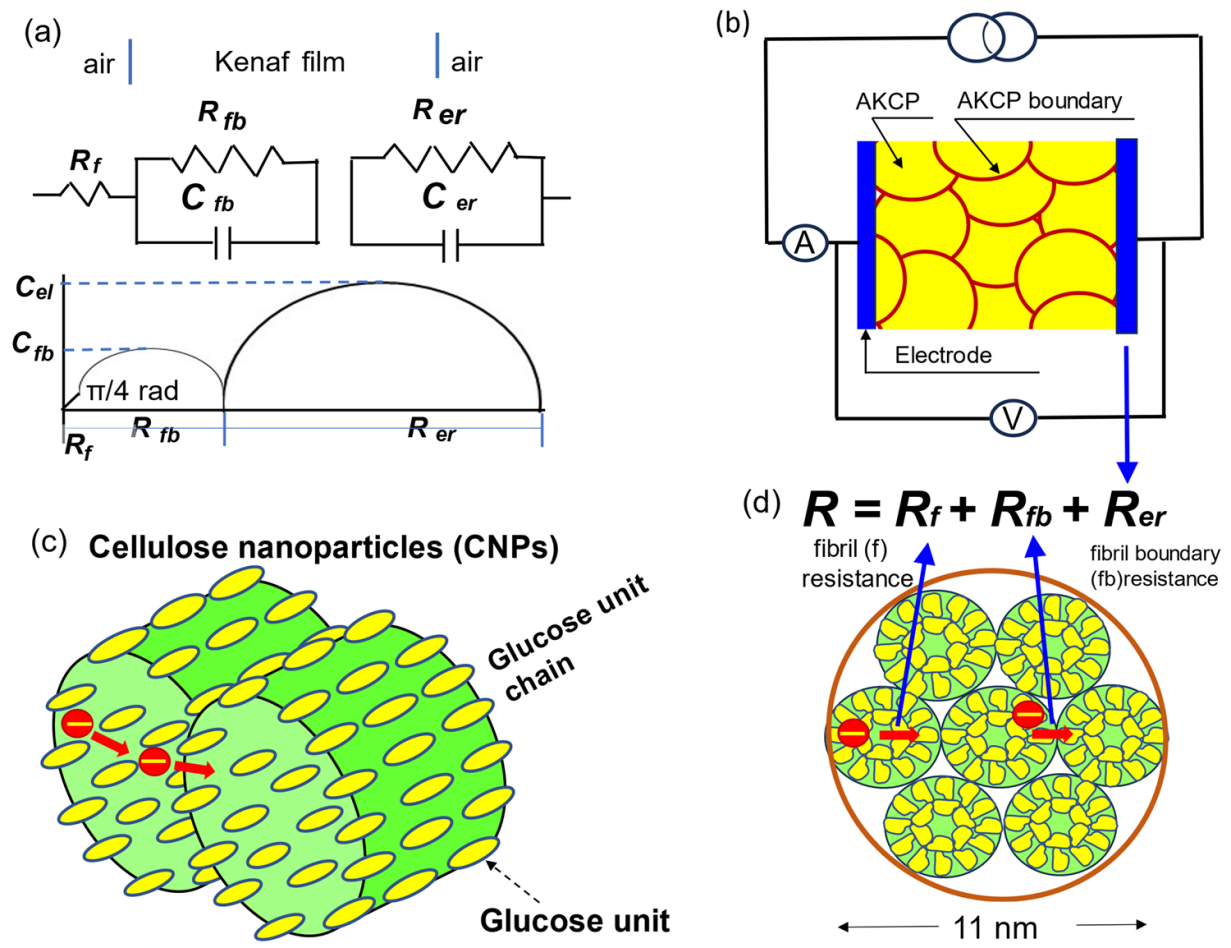
(**a**) Equivalent circuit corresponding to the Nyquist diagram shown in Fig. 3a,b. (**b**) Image of a semiconductor composed of AKCPs and their boundaries measured via the direct current method. (**c**) Image figure of cellulose nanoparticles. (**d**) Equivalent circuit for semiconductor conduction comprising fibril resistance *R*_*f*_, boundary resistance *R*_*b*_ and electrode interface resistance *R*_*er*_.

Based on the Nyquist diagram (Fig. 3a) and Debye relaxation peak in the Bode diagram (Fig. 3c), the equivalent electrical circuit of the AKCP can be regarded as a series coupling of three equivalent parallel circuits, as shown in Fig. 4a. Because the sample used in this study consists of 18 nanofibrils and their boundaries, the total resistance of the sample including the electrodes (Fig. 4b) is the resistance *R*_*f*_ of the nanofibrils in the AKCP, the boundary resistance *R*_*fb*_ between the AKCPs (Fig. 4c), and the electrode interface resistance *R*_*er*_ (Fig. 4d). The capacitance* C*_*f*_ of the nanofibrils in the AKCPs, the boundary capacitance *C*_*fb*_ between the AKCPs, and the electrode interface resistance *C*_*er*_ shown in Fig. 4a also play an important role in determining the semiconductor properties in this study. However, because the bonds between nanofibrils and the structure of AKCPs are unknown, precise structural analysis using soft X-rays is required.

## Conclusion

If cellulose, the most abundant natural renewable compound, is found to exhibit semiconductor properties, it can be used to develop many applications. In the present study, we reported the rectification effect of* n*-type semiconductors with N- and S-type negative resistivity properties derived from the glycosidic bond, C_1_–O^·^–C_4_, between two glucose units and O_1_ of cellulose molecules in AKCPs. The electron mobility was found to be 10.66 cm^2^/Vs, which is two orders of magnitude higher than that of conventional polymer semiconductors. The N- and S-type negative resistance effects may open up new fields in place of conventional *p–n* junction devices. We are working on conifers and hardwoods with the aim of expanding into areas different from conventionally engineered semiconductors.

## Methods

The AKCP specimen was fabricated on an Si substrate via spin coating, which was performed at a speed of 400 rpm for 5 s using a 2% (w/v) AKCP/water dispersion. The AKCP films were dried in a ventilated oven at 363 K. The specimens (12 mm wide, 14–101 μm thick, and 15 mm long) were mechanically sandwiched between an Al electrode and carbon electrode on the AKCPs (SI, Fig. S4). ESR measurements were performed at 295 K using a Q–band ESR spectrometer. Hall measurements were performed at 298 K using the conventional Van der Pauw technique. The current*–*voltage (*I–V*) and resistivity*–*voltage (*R–V*) characteristics were measured within 30 min after sample preparation and under DC voltages ranging from *–*210 to 210 V in air at a sweep rate of 51.4 V/s, using a Precision Source/Measure Unit (B2911A, Agilent). The AC impedance and frequency were measured using a potentiostat/galvanostat (SP-150, BioLogic Science). I–V measurements were performed using a Precision Source/Measure Unit (B2911A, Agilent).

### Supplementary Information

Supplementary Information.

## Data Availability

The authors declare that the data supporting the findings of this study are available within the paper, its supplementary information files, and from the National Tibetan Plateau Data Centre (https://doi.org/10.11888/Cryos.tpdc.272747).

## References

[1] MacDiarmid AG (2001). “Synthetic metals”: A novel role for organic polymers (Nobel Lecture). Angew. Chem. Int. Ed. Engl..

[2] Babel A, Jenekhe SA (2003). High electron mobility in ladder polymer field-effect transistors. J. Am. Chem. Soc..

[3] Elschner A, Kirchmeyer S, Lovenich W, Merker U, Reuter K (2010). PEDOT: Principles and Applications of an Intrinsically Conductive Polymer.

[4] Locklin J, Bao Z (2007). Organic Field-Effect Transistors.

[5] Coskun H (2020). Cofunction of protons as dopant and reactant activate the electrocatalytic hydrogen evolution in emeraldine-polyguanine. Adv. Mater. Interfaces.

[6] Fukuhara M (2022). A novel n-type semiconducting biomaterial. Sci. Rep..

[7] Nakagawa Y (2019). Structure/function correlation of thixotropic additives based on three leaf-like triamide derivatives containing three alkyl-chains. Coll. Surf. A.

[8] Sonntag C, Tipson RS, Horton D (1980). Free-radical reactions of carbohydrates as studied by radiation techniques. Advances in Carbohydrate Chemistry and Biochemistry.

[9] Swartz HM, Bolton JR, Borg DC (1972). Biological Applications of Electron Spin Resonance.

[10] Kim JU, Seo SG, Rogers JA (2023). Compound semiconductor devices for the skin. Nat. Mat..

[11] Yang L (2023). Achieving tissue-level softness on stretchable electronics through a generalizable soft interlayer design. Nat. Comm..

[12] Vébraité I, Hanein Y (2022). In the eye of the storm: Bi-directional electrophysiological investigation of the intact retina. Front. Neurosci..

[13] Fukuhara M, Yoshida H, Koyama K, Inoue A, Miura Y (2010). Electronic transport behaviors of Ni-Nb-Zr-H glassy alloys. J. Appl. Phys..

[14] Sawano F (2005). An organic thyristor. Nature.

[15] Suga H, Kawabata K, Yano M, Tanaka M (2017). Revised and Enlarged Visual Explanation, Electron Devices.

[16] Wei X, Yuan ZH (2010). Electronic transport behavior of diameter-graded Ag nanowires. Phys. Lett. A.

[17] Fukuhara M (2021). Amorphous cellulose nanofiber supercapacitors. Sci. Rep..

[18] Fukuhara M (2022). Amorphous cellulose nanofiber supercapacitors with voltage-charging performance. Sci. Rep..

[19] Fukuhara M, Yokotsuka T, Hashida T, Yamaguch K, Fujima N (2023). Amorphous alumina supercapacitors with voltage-charging performance. Europhys. Lett..

[20] Wadsworth A (2020). Modification of indacenodithiophene-based polymers and its impact on charge carrier mobility in organic thin-film transistors. J. Am. Chem. Soc..

[21] Takegami Y, Imamura S, Masuda F, Watanabe Y (1969). ESR spectra of alkoxy radicals and peroxy radicals related to oxidation reactions. J. Soc. Chem. Ind. Jpn..

[22] Yamauchi Y, Sugito M, Kuzuya M (1999). Plasma-induced free radicals of polycrystalline monocarbohydrates studied by electron spin resonance. Chem. Pharm. Bull..

[23] Li Q, Renneckar S (2011). Supramolecular structure characterization of molecularity thin cellulose I nanoparticles. Biomacromolecules.

[24] Shirakawa H, Louis EJ, MacDiarmid AG, Chiang CK, Heeger AJ (1977). Synthesis of electrically conducting organic polymers: Halogen derivatives of polyacetylene, (CH)x. J. Chem. Soc. Chem. Commun..

[25] Venkateshvaran D (2014). Approaching disorder-free transport in high-mobility conjugated polymers. Nature.

[26] Mikie T (2021). Extended π-electron dolocalization in quinoid-based conjugated polymers boosts intrachain charge carrier transport. Chem. Mater..

[27] Kurosawa T (2021). Chrysenodithiophene-based conjugated polymer: An elongated fused π-electronic backbone with a unique orbital structure toward efficient intermolecular carrier transport. Macromolecules.

[28] Farka D (2017). Anderson-localization and the Mott-Ioffe-Regel limit in glassy-metallic PEDOT. Adv. Electron. Mater..

[29] Kötz R, Carlen M (2000). Principles and applications of electrochemical capacitors. Electrochimica Acta.

[30] Itagaki M (2014). Electrochemistry, Impedance Method.

[31] Lee C (2014). Kenaf, Center for Crop Diversification.

[32] Muto T, Sugihara M, Goto T, Machi Y (1986). Electronic Materials Devices.

