# Supplementary material for: Radical electron-induced cellulose-semiconductors
Source: Sci Rep. 2024 Apr 15;14:8692. doi: 10.1038/s41598-024-59499-1 (PMC11018785; doi:10.1038/s41598-024-59499-1)
Supplement: Supplementary file 1 — Supplementary Information. [file 41598_2024_59499_MOESM1_ESM.pdf]

Supplementary Information

Radical Electron-Induced Cellulose-Semiconductors

Mikio Fukuhara,[1*] Tomonori Yokotsuka,[1] Tetsuo Samoto, [1] Masahiko Kumadaki,[2] Mitsuhiro Takeda,[3] and Toshiyuki Hashida[1]

[1] *New Industry Creation Hatchery Centre, Tohoku University, Aoba, Sendai 980-8579, Japan*

[2] *Technical Section, Semilab Japan, Yokohama, Japan. 222-0033*

[3] *National Institute of Technology, Sendai College, Natori, Japan 981-1239,*

S1. Methods

Kenaf is an annual herbaceous plant belonging to the mallow family, and it is being studied as a contributor to atmospheric decarbonisation[33-34]. Dried bast kenaf pulp fibres (harvested in Bangladesh, Toho Tokushu Pulp Corp. Kitakami, Japan) were retted in a 2.6 % water solution for 18 ks. Subsequently they were then defibrated using a cutter mixer for 60 s in air. Subsequently, they were miniaturised four times by roll milling in an aqueous solution. The precipitates were centrifuged in 50% water solution using a Fermix dispersion machine (30 L, Primix, Corp. Awaji, Japan) at a peripheral speed of 30 ml/s for 30 s, and supernatants were collected. Thirty milligrams of AKCP sample was completely dissolved in 4 ml of HNO_3 solution. Concentrations of 40 elements were measured using an inductively coupled plasma (ICP) analyzer (8800 triple-quadrupole ICP-MS, Agilent Technologies, Tokyo, Japan). The structure and angular spectral

diagram of the cellulose nano fibre below the transparent film surface were observed using confocal scanning microscopy (OPTELICS HYBRID+, Lasertec, Japan). The depth from the surface was calculated using z-scale values at the peak intensity positions of the interference fringes generated by white light and the two-beam interference objective lens. Attenuated Total Reflection Transmission FT-IR spectra for AKCP film with thickness of approximately 5 μm were collected at 298 K over the 4000–550 cm^{-1} regions, with a resolution of 4 cm^{-1}, using a JASCO model FT/IR 6300 spectrometer. For each sample, 100 scans were used for FT-IR. ESR measurements were performed at room temperature with a Q-band ESR spectrometer (JES-X330, JEOL) [power: 10 mW, modulation width: 2.0 mT, timer constant:0.1 s, sweep time: 60 s] at 298 and 103 K. Subsequently, g-values were measured relative to the fourth signal from the lower magnetic field (g =1.981) of Mn^{2+} in MgO. Hall measurements were performed in an AC magnetic field of 2.5 Tpk-pk with magnetic rotation speeds of 1 or 2 rpm for 10 V at 298 K using the conventional Van der Pauw technique with samples on an Si substrate, using a Hall effect measurement system (PDL-1000, SEMILAB). The sample structure was analysed through X-ray diffraction (XRD) in the reflection mode with monochromatic Cu Kα radiation. Selected-area electron diffraction (SAED) measurements were performed using a transmission electron microscopy (JEM-2100; JEOL). Surface morphologies were analysed using atomic force microscope (NanoScope V/Dimension Icon, Bruker AXS). All electronic measurements were performed in an Al shield box to prevent the results from being affected by electromagnetic interference from surroundings.

S2. Mineral contents

The problem of minerals being absorbed from soil through water is an important concern

for semiconductor properties. Therefore, the contents of forty elements in AKCP were determined ICP analysis. Table S1 shows the mass contents of these mineral elements. Although the amount of minerals in AKCP is extremely small, it is currently unknown whether these amounts affect semiconductor properties or not.

Table S1 ICP results of AKCP.

ppm

Li	B	Na	Mg	Al	Si	P	S	K	Ca
–	–	428.34	220.85	–	2231.35	–	–	112.41	–
Ti	V	Cr	Mn	Fe	Co	Ni	Cu	Zn	Ga
70.81	–	6.02	4.48	108.85	0.19	–	18.94	21.78	1.10
Ge	As	Zr	Nb	Mo	Ru	Rh	Pd	Ag	Cd
–	–	–	–	–	–	0.60	–	1.39	–
Sn	Sb	Te	Ba	La	Ce	Pr	Nd	Sm	Eu
–	–	–	2.08	0.40	0.16	0.22	–	–	–

S3. Effects of electron irradiation on AKCP

In electron observations of CNFs made from Varonia, Cotton, Ramie, wood, and Acetobacter cellulose, damage has been reported when the electron dose to the specimen exceeds approximately 3×10^{20} e/m^2 at an accelerated voltage of 200 kV[35]. In this study, AKCP samples were examined for electron irradiation damage by electron diffraction for 0, 600, and 1,800 s of irradiation at 100 kV with a dose rate of 1.16×10^{25} e/m^{2}s . The SAED patterns are shown in Fig. S1. No degradation was observed during irradiation at 100 kV. The halo Debye rings were slightly blurred after 1,800 s of irradiation but did not decompose or disappear.

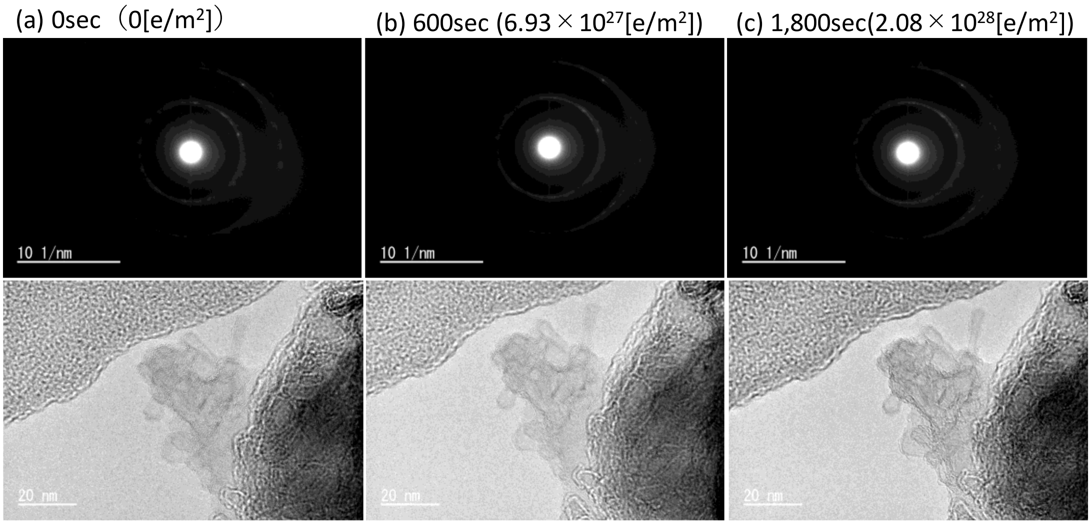

Fig. S1 SAED patterns of AKCP irradiated at 100 kV with 1.16×10^{25} e/m^{2}s for 0, 600, and

S4. Structural morphology characterised via white interferometer microscopy (WIM), transmission electron microscopy (TEM) and atomic force microscopy (AFM).

The structural morphology of the AKCP samples was investigated. Figure S2a and S2b illustrate an internal microstructure and angular spectra diagram of the distribution of grain shape orientations at 2 μm below the transparent surface of the AKCP, which were obtained using WIM. The angular spectrum is oriented in almost all directions, indicating defibrillated particles. However, internal angular spectral diagram (Fig. S2d) of the microstructure (Fig. S2c) of the AKCF, which was used as a comparison is fibrous with a clear orientation and non-defibrillated particles. The wide-field X-ray analysis pattern (Fig. S2e) comprises amorphous cellulose, and it is characterised by three broad peaks at approximately 16°, 23°, and 70°[36]. Figure S2f shows a scanning electron microscopy (SEM) photograph at 5 keV. The surface contains particulate aggregates of approximately

50 nm. Fig. S2g shows an AFM photograph comprising an average particle size of 11 nm. Figure S2h displays a TEM image of the sample observed at 100 keV. It is a microstructural photograph of entangled cellulose bundles with a diameter of approximately 4 nm. The selected area electron diffraction (SAED) pattern of irregularly arranged nanofibrils obtained from the entire field is shown in Fig. S2h. The SAED patterns show diffuse spots and hollow Debye rings, indicating a mixture of amorphous and nanocrystalline phases with a small degree of crystallinity. The cellulose nanofibril containing nanocrystals surrounding 18 glucose units composed of the amorphous phase

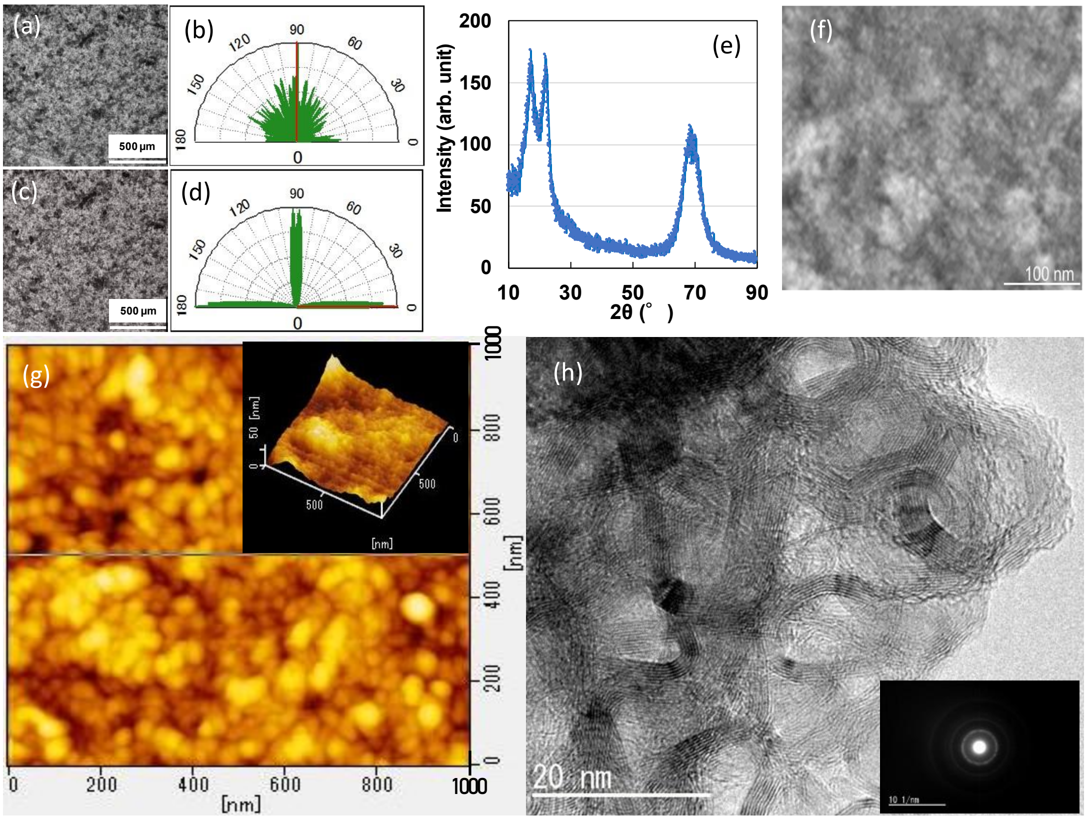

Fig. S2 Internal microstructures (a) and (c) and angular spectra (b) and (d) at 2 μm below the transparent surface of AKCP as determined via WIM. XRD pattern (e), SEM image (f) at 5 keV, AFM image (g) at 100 keV, and TEM image (h) at 100 keV of the AKCP surface. Insets in (g) and (h) show the three-dimensional AFM and SAED pattern, respectively.

can be inferred from the amorphous XRD pattern shown in Fig. S2e, the Nyquist diagram shown in Fig. 3b, and the amorphous phase in Fig. S4. Furthermore, the samples used in this study may exist as clusters composed of cellulose, as inferred from the amorphous alloys composed of dodecahedral, icosahedral [37-39], C_{60} [40] and icosahedral $(H_2O)_{280}$ water clusters[41].

S5. TEM image and SAED pattern of nanofibril phases

The TEM image and SAED pattern for the outside regions of the nanofibril phase, which makes up the majority of the tissue, are illustrated in Fig. S3, depicting a completely amorphous hollow pattern.

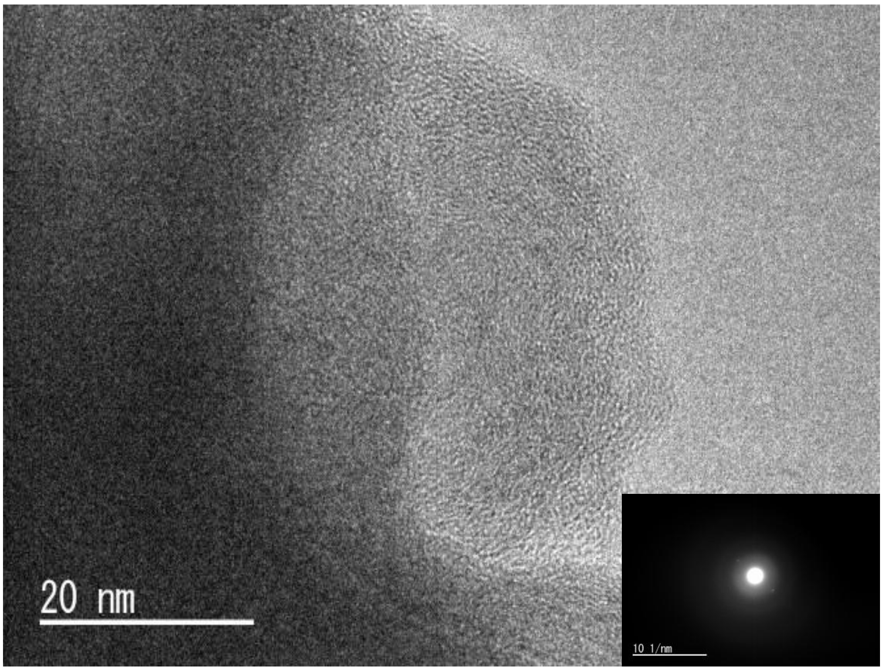

Fig. S3 TEM image and SAED pattern of the amorphous phase.

S6. Depth analysis of p-Si substrate under ACSP film

The nature of the interface between the n-AKCP/p-Si and the p-Si substrate to which the AKCP film is bonded depends on the oxidation state of the p-Si at this interface. If it is not oxidised, it forms a p-n junction; if it is oxidized, it is determined to be a Schottky junction. To determine it, the depth dependence of Si and O in the p-Si depth direction was measured. The depth profiles of the Si and O elements and the profiles of the Si 2*p* and O 1*s* spectra are illustrated in Fig. S4(a), (b), and (c). The depth dependence of these concentration files indicates that the oxide layer is approximately 5 nm deep. Two profiles of the Si 2*p* and O 1*s* spectra identify the composition of SiO_2[42]. Because the normal

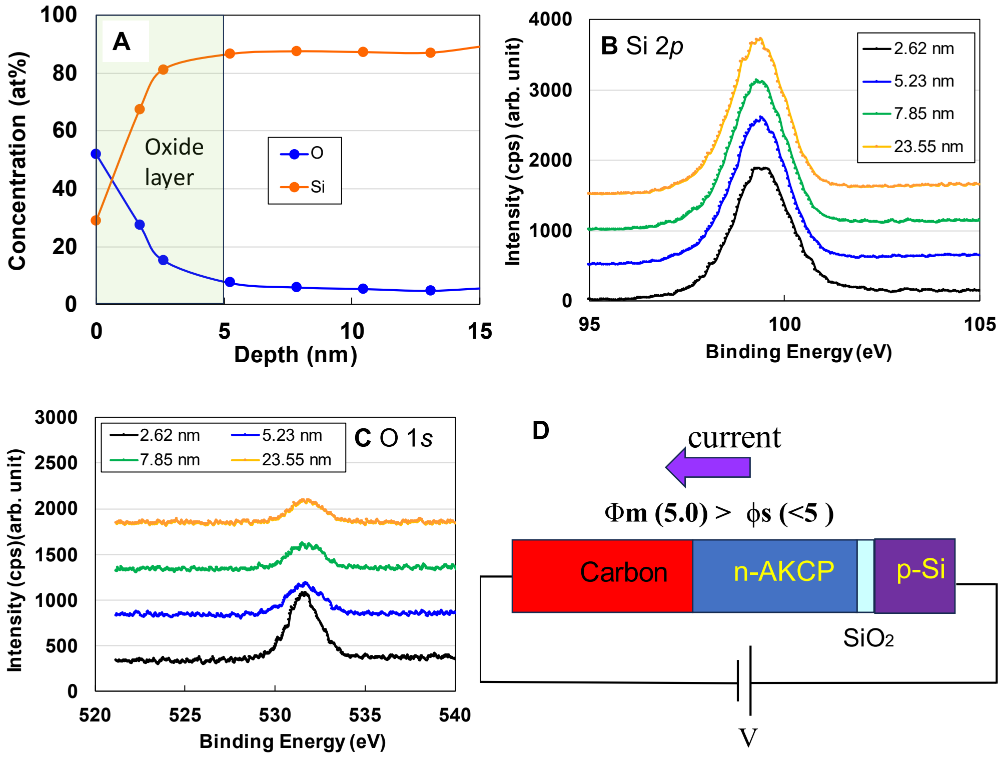

fig. S4(A) Depth profile of Si and O elements. (B) Profiles of Si 2*p* and O 1*s* (C) spectra. (D) Device model used in this study.

operation of a MOSFET with a gate oxide thickness of 1.5 nm has been confirmed[43], it can be estimated as an SiO_2 oxide layer for the experiments performed in this study. Thus,

the device used in this experiment was considered to have a Schottky coupling (Fig. S4(d)) between carbon with a work function of 5 and n-AKCP with a work function of 4.77[18].

S7. Consideration of radicals on cellulose molecule

The effect of one-sidedness, indicated by the difference in the electronegativity of the atoms in a compound owing to the ease with which the atoms attract or release electrons, is called the induced effect and is a guide to organic radical generation. In cellulose $(C_6H_{10}O_5)_n$, when comparing the electronegativity of O_5 and O_1, the electrons in O_5 are biased towards C_1, as shown in Fig. 2d, primarily because the electronegativity of O_1 between the two glucose units is greater than that of O_5. Thus, one glucose unit becomes an electron-withdrawing group because the electronegativities of C, H, and O are 2.55, 2.20 and 3.44, respectively, and O_1 is biased towards electrons with an electronegativity of 4.26. On the other hand, electrons are biased towards O_2 and O_3 with an electronegativity of 2.48 and towards O_6 with an electronegativity of 2.29. Therefore, most atoms are biased towards O_1 in C_1-O_1. Consequently, an electron radical is induced in O_1. Radicals formed on the alkoxyl groups of side chains, such as positions C_1 and C_2, are more reactive than radicals on the glucose units of the main chain, and they cannot be C-O$^{\bullet}$ radicals because they quickly proceed to secondary reactions such as subsequent rearrangement and recombination. On the other hand, the radical formed at position C-6 is a secondary radical, which is unstable and therefore preferred for the rapid progress of cross-linking, but can be excluded from consideration of the radical formation mechanism. Thus, the radical electrons are derived from the glycosidic bond, C_1- O$^{\bullet}$-C_4, between the

two glucose units and O_1, of cellulose molecules.

S8. Other *I−V* characteristics

Visible oscillation phenomena can be observed in the *I*–*V* curves when the thickness of the device exceeds 50 μm, as shown in the AKCP device with a thickness of 66 μm (Fig. S5a). Frequency analysis of the curve reveals a peak at 110.5 Hz (Fig. S5b). The peak position as a function of thickness is shown in Fig. S5c. The peak frequency decreases as

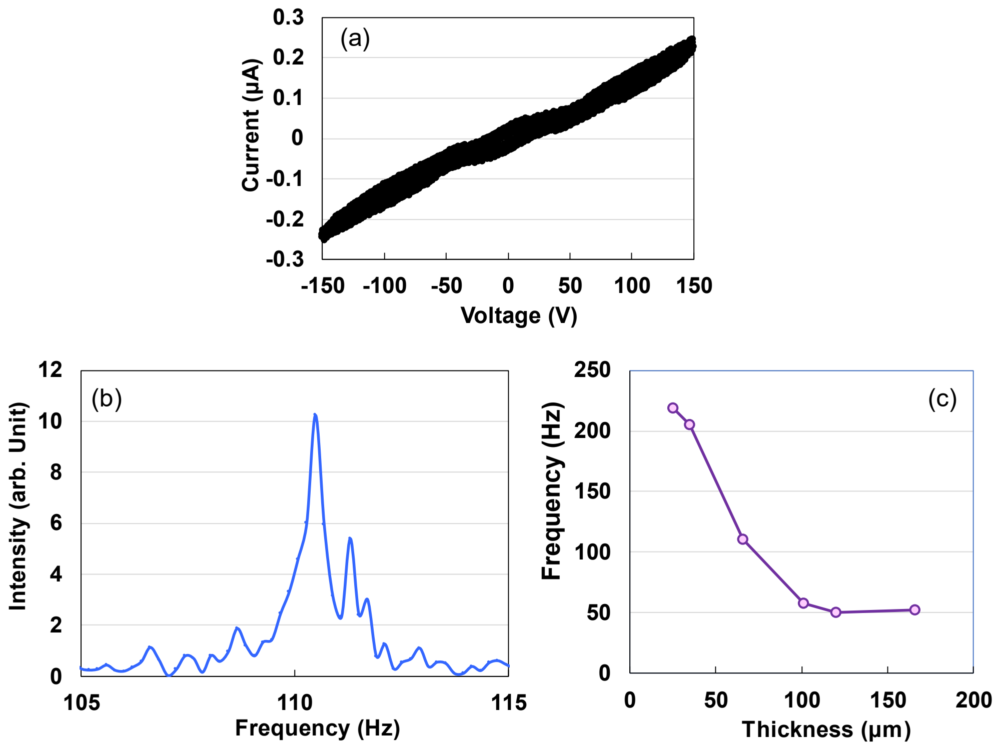

Fig. S5 (a) Voltage-controlled *I*–*V* characteristics of AKCP with a thickness of 66 μm at a sweep rate of 51.5 V/s. (b) Frequency analysis, (c) The peak position as a function of thickness.

the thickness increases, and it tends to be a constant thickness exceeding 120 μm. This behaviour would be attributed to capacitance.

S9. Conduction mechanism

Negative-resistance devices have a differential resistance defined as R = d*V*/d*I* < 0. They can be classified into static negative resistance[44], where the negative resistance characteristic appears on the DC *I-V* characteristic, and dynamic negative resistance, where the negative resistance characteristic does not appear on the DC *I-V* characteristic but shows negative resistance owing to effects such as carrier travel time. Static negative-resistance devices can be explained in terms of *pn* junction theory, such as tunnel diodes, thyristors, and junk-shot transistors. Dynamic negative-resistance devices, such as impact avalanche transit time (IMPATT) and Gunn diodes, can be explained by the carrier travel time and peculiarities of the band structure of the material. The bio-semiconductor phenomenon in this study is not caused by a *pn* junction but by a Schottky junction. This means that the phenomenon is induced by the electron avalanche and the carrier travelling speed.

Systems that exhibit differential negative resistance can be divided into two classes: voltage-controlled (N-type) and current-controlled (S-type)[45]. The mechanisms causing these negative resistances can be divided into three broad categories. (1) processes caused by the Joule heating of conduction electrons, which causes changes in their number or mobility, (2) processes inspired by special semi-permanent space charge distributions, and (3) processes caused by phase changes or atomic arrangements in the host insulator. The semiconducting properties in this study are involved in the second category, as inferred from the organic-induced electrons in Fig. 2(a), the dielectric characteristics in Figs. 3(b) and 3(c), and the cellulose molecular model in Fig. 4(a). Models involving this particular space-charge distribution include the impurity band theory of Hickmott for

impurity bands between insulator band gaps[46], the electron hopping conduction theory of Simmons and Verderber[47], the space-charge-limited ionic current theory of Barriac et al.[48], and Mott's electron-hole tunnelling theory in Schottky barriers[49]. To open up new fields of electronics in bio-semiconductors, the conduction mechanism of semiconductor properties must be clarified. This clarification is left for future research.

References

33. Tahir, P. Md., Ahmed, A. B., Saiful, Azry, S.O. A. & Ahmed, Z. Retting process of some bast plant fibres and its effect on fibre quality: a review, *Bioresources*, **6**, 5260–5281 (2011). 10.15376/biores.6.4.5260-5281

34. Lam, T. B. T., Hori, K. & Iiyama, K. Structural characteristics of cell walls of kenaf (Hibiscus cannabinus L.) and fixation of carbon dioxide. *J. Wood Sci*. **49**, 255–261 (2003). 10.1007/s10086-002-0469-7.

35. Sugiyama, J. Harada, H. Fujiyoshi, Y. & Ueda, N. Lattice images of cellulose crystallites, *Electron Microscope*, **20**, 143−147 (1985).

36. Kim, D-Y., Lee, B.-M., Koo, D. H., Kang, P.-H. & Jeum, J. P., Preparation of nanocellulose from a kenaf core using E-beam irradiation and acid hydrolysis, *Cellulose*, **23**, 3039–3049 (2016). 10.1007/s10570-016-1037-4

37. Takagi, T. et al. Local structure of amorphous $Zr_{70}Pd_{30}$ alloy studied by electron diffraction. *Appl. Phys. Lett*. **79**, 485–487 (2001). 10.1063/1.1383055.

38. Fujima, N, Hara, K., Hoshino, T. & Fukuhara, M. Structural and electronic properties of $Ni_5Nb_3Zr_5$ clusters as a local structural unit of Ni-Nb-Zr glassy alloys. *Eur. Phys. J. D* **63**, 177–181 (2011). 10.1140/epjd/e2011-10514-9.

39. Fukuhara, M. Electronic Properties of Nanoclusters in Amorphous Materials, (Cambridge Scholars Pub., New Castle upon Tyne, UK, 2019). ISBN (10):1-5275-3755-2.

40. Shibuya, T. & Yoshitani, M. Two icosahedral structures for the C_{60} cluster, *Chem. Phys. Lett.* 137, 13–16 (1987). 10.1016/0009-2614(87)80295-6

41. Chaplin, M. Water structure and science. https://water.lsbu.ac.uk/water/icosahedral water clusters.html. 10.1021/acs.macromol.0c00984.

42. Wagner, C. H. The NIST X-ray Photoelectron Spectroscopy (XPS) Database, NIST Technical Note1289 (NIST Publications 1991).

43. Momose, H. S. et al., Tunneling gate oxide approach to ultra-high current drive in small-geometry MOSFETs, *IEDM Tech. Dig.* **94**, 593−596 (1994).

44. Dearnaley, G., Stoneham, A. M. & Morgan, D. V. Electrical phenomena in amorphous oxide films, *Rep. Prog. Phys.*, **33**, 1129−1191 (1970).

45. Simmons, J. G., Blood, P., Orton, J. W. & Lawless, K. R. Electrical phenomena in amorphous oxide films, *Rep. Prog. Phys.* 33, 1129−1191 (1970).

46. Hickmott, T. W. Low-Frequency Negative Resistance in Thin Anodic Oxide Films. *J. Appl. Phys*., **33**, 2669–2682 (1962). 10.1063/1.1702530.

47. Simmons, J. G. & Verderber, R. R., New conduction and reversible memory phenomena in thin insulating films, *Proc. R. Soc*. **A301**, 77−102 (1967). 10.1098/rspa.1967.0191.

48. Barriac, C., Pinard, P. & Davoine, F. Étude des propriétés électriques des structures Al–Al_2O_3–metal, *Phys. Stat. Solidi*. **34**, 621−633 (1969).

49. Mott, N. F. Conduction and switching in non-crystalline materials. *Contemp. Phys*. **10**, 125−138 (1969). 10.1080/00107516908220104
